# Genetic Study for Identifying Beta Thalassemia Trait in Relatives of Children with Beta Thalassemia Major

**DOI:** 10.7759/cureus.70251

**Published:** 2024-09-26

**Authors:** Osama M Elasheer, Shimaa M Radi, Mostafa S Khalaf, Mohamed H Ghazally, Dalia A Nigm, Mostafa M Embaby

**Affiliations:** 1 Pediatrics, Assiut University, Assiut, EGY; 2 Clinical Pathology, Assiut University, Assiut, EGY

**Keywords:** familial trait, gene study, high-performance liquid chromatography (hplc), thalassemia carrier, thalassemia minor, thalassemia trait

## Abstract

Background: The most common inherited illness, thalassemia, is thought to have a detrimental effect on public health, particularly in endemic areas. Children with beta thalassemia disease have several mutations. Prevention and premarital examination are still the most effective measures to lessen the burden of beta thalassemia.

Objectives: This study primarily aimed to determine the beta thalassemia carriers in relatives of beta thalassemia major children, the role of gene study in the confirmation of beta thalassemia trait diagnosis, and to detect the genetic defect in the relatives of beta thalassemia major children.

Materials and methods: The cross-sectional study was conducted on 109 healthy children, aged between six months and 18 years, who were the relatives (second and fourth degree) of beta thalassemia major cases.

Results: Gene screening, using the amplification refractory mutation system (ARMS) polymerase chain reaction (PCR), covered the most common 22 alleles in the Mediterranean region, and was successful in detecting 61.5% of beta-globin chain mutations of studied participants, in addition to high prevalence (34.8%) of beta thalassemia carriers among the relatives of beta thalassemia children.

Conclusion: The beta thalassemia carrier rate was found to be highly prevalent among relatives of beta thalassemia major children. Despite the accuracy of gene screening in the detection of beta thalassemia carriers, the use of the most common 22 alleles can only detect 61.5% of carriers; hence, the value of tested gene study is still limited in the detection of carrier rates.

## Introduction

Beta thalassemia major is a severe, life-limiting illness that profoundly impairs social and intellectual functioning. Due to doctor's appointments, hospital stays for monthly blood transfusions, and/or issue therapy, children frequently have to miss school. They are increasingly dependent on others and may have some psychological problems [1].

The high prevalence of beta thalassemia carriers (34.9%) and the rise in newly diagnosed cases underscore the critical need for developing a preventive program for the disease in Egypt [2]. People can discover more about their potential and present health, as well as the health of their progeny, through genetic disease screening. The most crucial actions to reduce the carrier rate of beta thalassemia are still preconceptions, orientation, and premarital testing [3].

Primers-specific amplification, amplification refractory mutation system (ARMS), and reverse oligonucleotide hybridization with oligonucleotide probes are the two most widely used methods for detecting known mutations based on polymerase chain reaction (PCR). If focused mutation analysis is not successful in identifying the mutation, analysis of the beta-globin gene sequence can be utilized to identify unidentified mutations [4].

Comprehensive national prevention programs that offer information on currently used tests for prenatal diagnosis, pre-implantation diagnosis, carrier screening, counseling, public awareness, and education have been established in a number of countries [5].

Egypt lacks a national thalassemia prevention program, despite the country's high carrier incidence and growing annual number of new cases. Few studies have been done to find the thalassemia carrier rate in Egypt [1]. One estimate puts the number of live births affected by thalassemia annually between 1,000 and 1.5 million [6]. As of 2021, the disease is present in 5.3-9% of people in Egypt. A cross-sectional study on 2118 relatives of thalassemia patients from various Egyptian governorates in the Mid Delta region showed that 53.02% had microcytic hypochromic anemia [7]. 

This study aimed to ascertain the frequency of beta thalassemia carriers among close relatives of beta thalassemia major patients, aged between six months and 18 years, determine how gene studies contribute to the confirmation of the beta thalassemia trait diagnosis, and detect the genetic defect in relatives of beta thalassemia major children.

## Materials and methods

Study design and subjects

This was a cross-sectional study conducted at the Assiut University Children's Hospital, Assiut, Egypt, from October 2019 to October 2021. The study was approved by the Ethics Committee of the Faculty of Medicine, Assiut University (approval number: 04-2023-10006). The trial was registered on clinicaltrial.gov (NCT03822585).

Inclusion and exclusion criteria

Second or fourth-degree relatives of children with beta thalassemia, aged between six months and 18 years were included in the study. Relatives known to be thalassemic, who have received a blood transfusion, or had any other chronic illness were excluded from the study. Before the trial began, the guardians of all the participants provided written informed consent.

Study process

**Figure 1:**
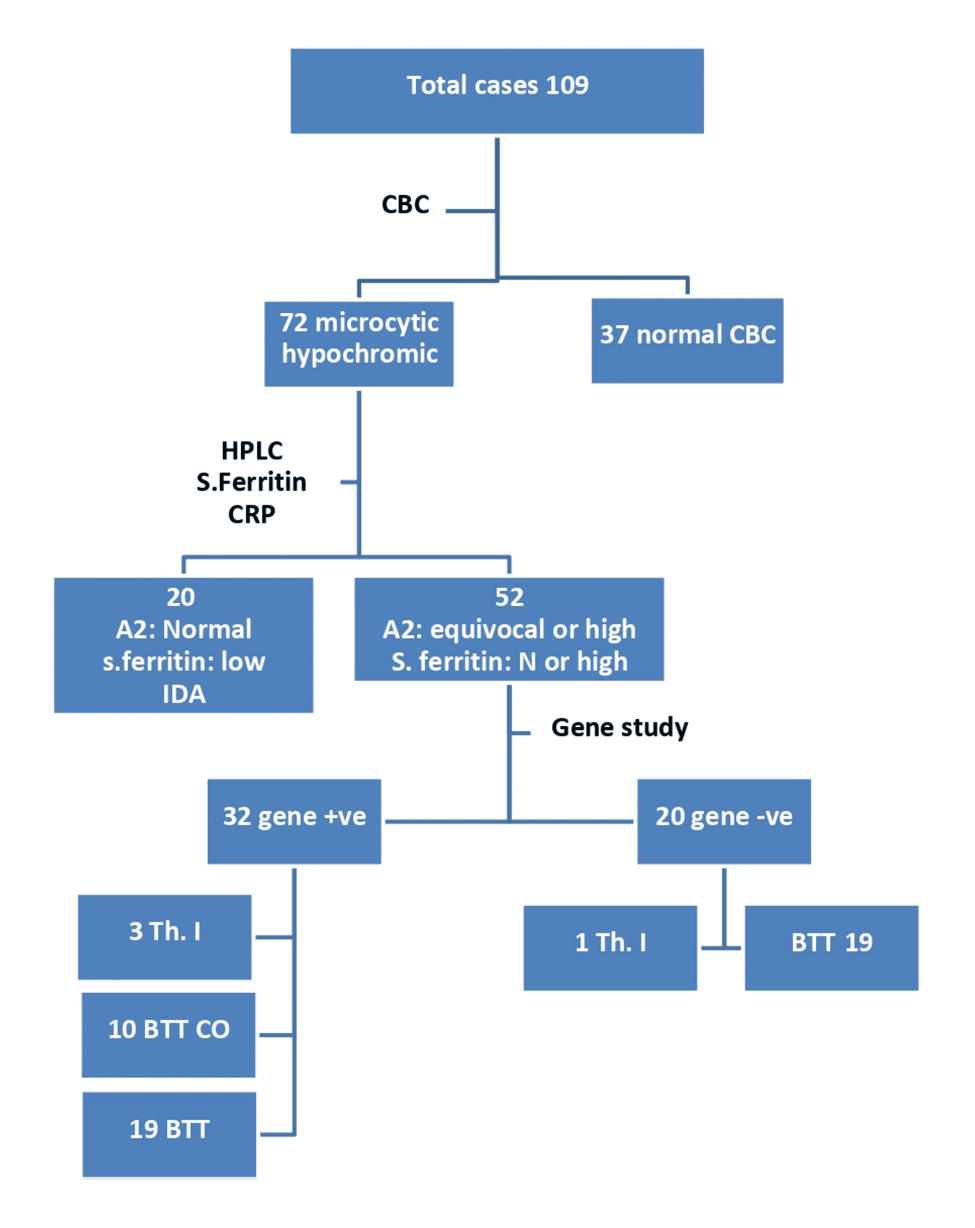
Flowchart of participants A2: hemoglobin A2; BTT: beta thalassemia trait; BTT_Co: beta thalassemia coexistent with iron deficiency anemia; CBC: complete blood count; CRP: C-reactive protein; HPLC: high-performance liquid chromatography; IDA: iron deficiency anaemia; N: normal, Th.I: thalassemia intermediate

The flowchart of the study process is shown in Figure 1. Cases were numbered according to their attendance at the clinic, and then a relative for every fifth case was recruited. The STROBE (Strengthening the Reporting of Observational Studies in Epidemiology) checklist and the Helsinki Declaration principles were followed during the study's execution [8,9].

Sample size calculation

G*Power 3 software (Heinrich-Heine-Universität Düsseldorf, Düsseldorf, Germany) was used to calculate the sample size. To find an effect size of 0.5, a minimum calculated sample of 105 healthy children was required. According to the beta thalassemia carrier rate, with an error probability of 5% and 95% power, a total of 109 children were included.

Technique

Study participants had the following investigations in addition to a complete physical examination and recording of their history: complete blood counts, serum ferritin, C-reactive protein (CRP), and high-performance liquid chromatography (HPLC) were done on the D10 automated hemoglobin testing system (Bio-Rad Laboratories, Inc., Hercules, California, United States). The analytes were separated chromatographically using ion exchange HPLC. The D-10 automatically diluted the samples before injecting them into the analytical cartridge. Hemoglobin was separated in the cartridge according to its ionic interactions with the material by use of a programmed buffer gradient of increasing ionic strength that is delivered by the D-10. After being separated, the hemoglobin travels through the filter photometer's flow cell, where variations in absorbance at 415 nm are detected for those who showed microcytic hypochromic anemia, and a gene study for those who showed high or equivocal HbA2 The gene study was done by B-Globin Strip Assay Med (ViennaLab Diagnostics GmbH, Vienna, Austria). The B-globin strip assay, which uses biotinylated primers for polymerase chain reaction amplification and hybridizes the resulting products to a test strip containing allele-specific oligonucleotide probes immobilized as a series of parallel lines, is based on the principle of reverse hybridization. Twenty-two of the most common beta-globin mutations in the Mediterranean region are covered by the assay [10], of which the ones detected in our study are shown in Table 1.

**Table 1:** Detected gene alleles in our studied cases IVS: intervening sequence; G: guanine; T: thymine; A: adenine; C: cytosine

	F	%
IVS 1.6 (T>C)	10	31.3%
IVS 1.110 (G>A)	7	21.9%
IVS 1.1 (G>A)	3	9.4%
IVS 2.848 (C>A)	3	9.4%
IVS 2.745 (C>G)	2	6.3%
CODON 39	2	6.3%
CODON 8 (-AA)	2	6.3%
-30 (T>A)	1	3.1%
-87 (C>G)	1	3.1%
CODON 5 (-CT)	1	3.1%
CODON 27 (G>T)	1	3.1%
IVS 2.1 (G>A)	1	3.1%
IVS 1.5 (T>C)	1	3.1%
CODON 44 (-C)	1	3.1%

Statistical analysis

IBM SPSS Statistics for Windows, Version 19.0 (Released 2010; IBM Corp., Armonk, New York, United States) [11] was used for statistical analysis. The statistical significance tests were ANOVA/Kruskal-Wallis and Chi-square for comparison of differences in means, medians, and frequencies in different groups. P-values of less than 0.05 were regarded as statistically significant. The Youden index is a single statistic that captures the performance of a dichotomous diagnostic test (Youden index = sensitivity + specificity - 1). It was determined using the Medcale program for Windows version 19.4 [12]. The biggest diagnostic cut-off point of the Youden index is used as the optimal cut-off value [13]. To shed light on the sociodemographic findings of the groups under study, an exact test was used to assess the frequency difference between the groups. The laboratory parameters of the groups under study were clarified through pairwise comparisons using the post-hoc test and Bonferroni correction, while the Kruskal-Wallis test was employed to compare the median difference between the groups.

## Results

A total of 109 relatives aged between six months and 18 years were included. Of these, 52 cases were positive for HbA2. Table 2 demonstrates the sociodemographic traits of these 52 children: 40.4% were males; most of them were from rural areas, with a P value of 0.0001. The history of consanguineous was significantly positive, with a P value of 0.006.

**Table 2:** Sociodemographic data of participants who were positive for HbA2 (N=52) IDA: iron deficiency anemia P-value was considered significant if <0.05

Characteristics	Intermediatte Thalessamia (n=4)	Beta Thalassemia Trait (n=38)	Beta Thalassemia with IDA (n=10)	P-value
Sex				0.144
Male	0 (0%)	19 (50%)	2 (20%)	
Female	4 (100%)	19 (50%)	8 (80%)	
Residence				0.001
Urban	0 (0%)	6 (15.8%)	0 (0%)	
Rural	4 (100%)	32 (84.2%)	10 (100%)	
Consanguinity				
Positive	4 (100%)	33 (86.8%)	9 (90%)	0.006
Negative	0 (0%)	5 (13.2%)	1 (10%)	

Table 3 shows the laboratory parameters among the participants positive for *HbA2*, where the red blood cell (RBC) count was found to be higher in the beta thalassemia trait (BTT) group with low mean corpuscular hemoglobin (MCH), red cell distribution width (RDW)%, red cell distribution width index (RDWI), and Mentzer index (MI). Meanwhile, relationships between other parameters (hemoglobin (Hb), mean corpuscular volume (MCV), platelets (PLT), and WBC (white blood cell) were insignificant.

**Table 3:** Laboratory parameters of participants positive for HbA2 (N=52) IQR: interquartile range; IDA: iron deficiency anemia; RBC: red blood cell; Hb: hemoglobin; MCV: mean corpuscular volume; MCH: mean corpuscular hemoglobin; RDW: red cell distribution width; RDWI: red cell distribution width index; MI: Mentzer index; Plt: platelets; WBC: white blood cell; CRP: C-reactive protein p-value was considered significant if <0.05; P value in horizontal lines demonstrates the significance between the two mentioned groups while that in vertical line presents the significance among the three studied groups.

Median (IQR)	Group 1, Intermediatte Thalessamia (n=4)	Group 2, Beta Thalassemia Trait (n=38)	Group 3, Beta Thalassemia with IDA (n=10)	P-value
RBC	4.8 (0.9)	5.7 (0.6)	5.1 (0.7)	< 0.001
P-value	Group 1 vs. Group 2=0.011	Group 2 vs. Group 3=0.604	Group 1 vs. Group 3=0.661	
HGB	9.7 (2)	10.5 (0.8)	10.7 (1.5)	0.044
P-value	Group 1 vs. Group 2=0.120	Group 2 vs. Group 3=0.802	Group 1 vs. Group 3=0.096	
MCV	67.4 (4.8)	61.1 (6.4)	67.6 (7.5)	0.058*
MCH	19.3 (2.3)	18.5 (1.4)	20.3 (5)	0.006
P-value	Group 1 vs. Group 2=0.237	Group 2 vs. Group 3=0.564	Group 1 vs. Group 3=0.769	
RDW%	23.6 (3.2)	15.6 (1.4)	15.7 (3)	0.002
P-value	Group 1 vs. Group 2=0.014	Group 2 vs. Group 3=0.942	Group 1 vs. Group 3=0.041	
RDWI	337 (75)	173 (31)	217 (73)	0.001
P-value	Group 1 vs. Group 2<0.001	Group 2 vs. Group 3=0.015	Group 1 vs. Group 3=0.123	
MI	14.1 (2.7)	10.8 (2.1)	13.3 (4.5)	0.001*
P-value	Group 1 vs. Group 2=0.029	Group 2 vs. Group 3=0.144	Group 1 vs. Group 3=0.524	
Platelet	349 (206)	333 (116)	381 (113)	0.272
WBCs	12.2 (2.5)	6.5 (3)	6.4 (4)	0.016
P-value	Group 1 vs. Group 2=0.010	Group 2 vs. Group 3=0.991	Group 1 vs. Group 3=0.030	
CRP	0 (0)	0 (6)	6 (12)	0.103
Ferritin	24 (15)	80 (70)	18 (10.5)	< 0.001
P-value	Group 1 vs. Group 2=0.037	Group 2 vs. Group 3<0.001	Group 1 vs. Group 3=0.246	

Table 4 illustrates the HPLC and thalassemia gene expression of the studied groups. All 52 cases (of the 72 cases of microcytosis, 52 cases were positive or equivocal for HbA2, which suggested possible BTT) were further subjected to gene study (the ARMS of the most common 22 alleles in the Mediterranean region for detection of beta-globin chain mutation of thalassemia). The genes responsible for thalassemia were positive in 61.5% of participants in the study.

**Table 4:** HPLC and thalassemia gene expression IDA: iron deficiency anemia; HgF: fetal hemoglobin; HPLC: high-performance liquid chromatography P-value was considered significant if <0.05 * Significance; ** Very strong significance; ***Extremely strong significance

					Intermediatte Thalessamia			Beta Thalassemia Trait			Beta Thalassemia with IDA		
		Frequency	Percentage	P-value	Frequency	Percentage	P-value	Frequency	Percentage	P=value	Frequency	Percentage	P-value
HbA2	Normal	26	36.1	0.016*	1	25	0.5	3	7.9	<0.001***	2	20	0.088
	High (>3.5%)	46	63.9		3	75		35	92.1		8	80	
HbF	Normal	64	88.9	0.001***	0	0		35	92.1	<0.001***	9	90	0.02*
	High (>2%)	8	11.1		4 (>10%)	100		3 (>2%)	7.9		1 (>2%)	10	
Gene	Positive	32	61.5	0.002**	3	75	0.5	19	50.0	0.129	10	100	
	Negative	20	38.5		1	25		19	50.0		0	0	

**Figure 2:**
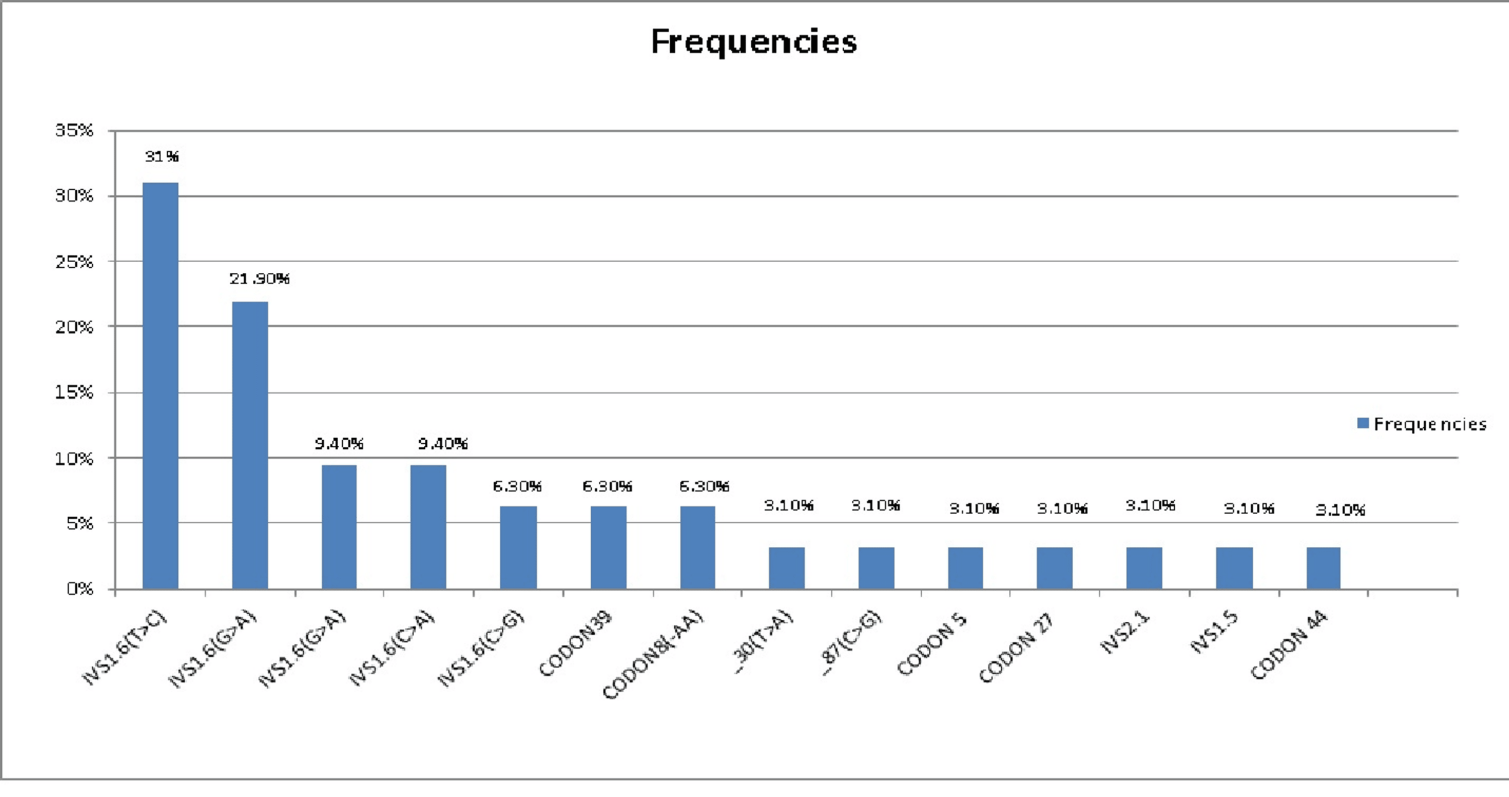
Frequency of genes.

Figure 2 shows the frequency of genes in the studied cases. Molecular analysis was successful in determining 32 participants (out of 52 carriers based on HPLC), where 14 mutations were detected from 22 beta-globin mutations, which are frequently found in the Mediterranean region. Out of 14 different mutations, the IVS 1.6 (T>C) mutation was the most prevalent one (31.3%), followed by IVS 1.110 (G>A) (21.9%); a homozygous mutation was found in 16 patients (50%) out of 32, heterozygous in 12 patients (37.5%), and compound mutations in four patients (12.5%). Twenty patients (38.5%) showed unknown mutations. Our study's approach had a detection rate of 63.6% (14 of the 22 alleles examined).

**Figure 3:**
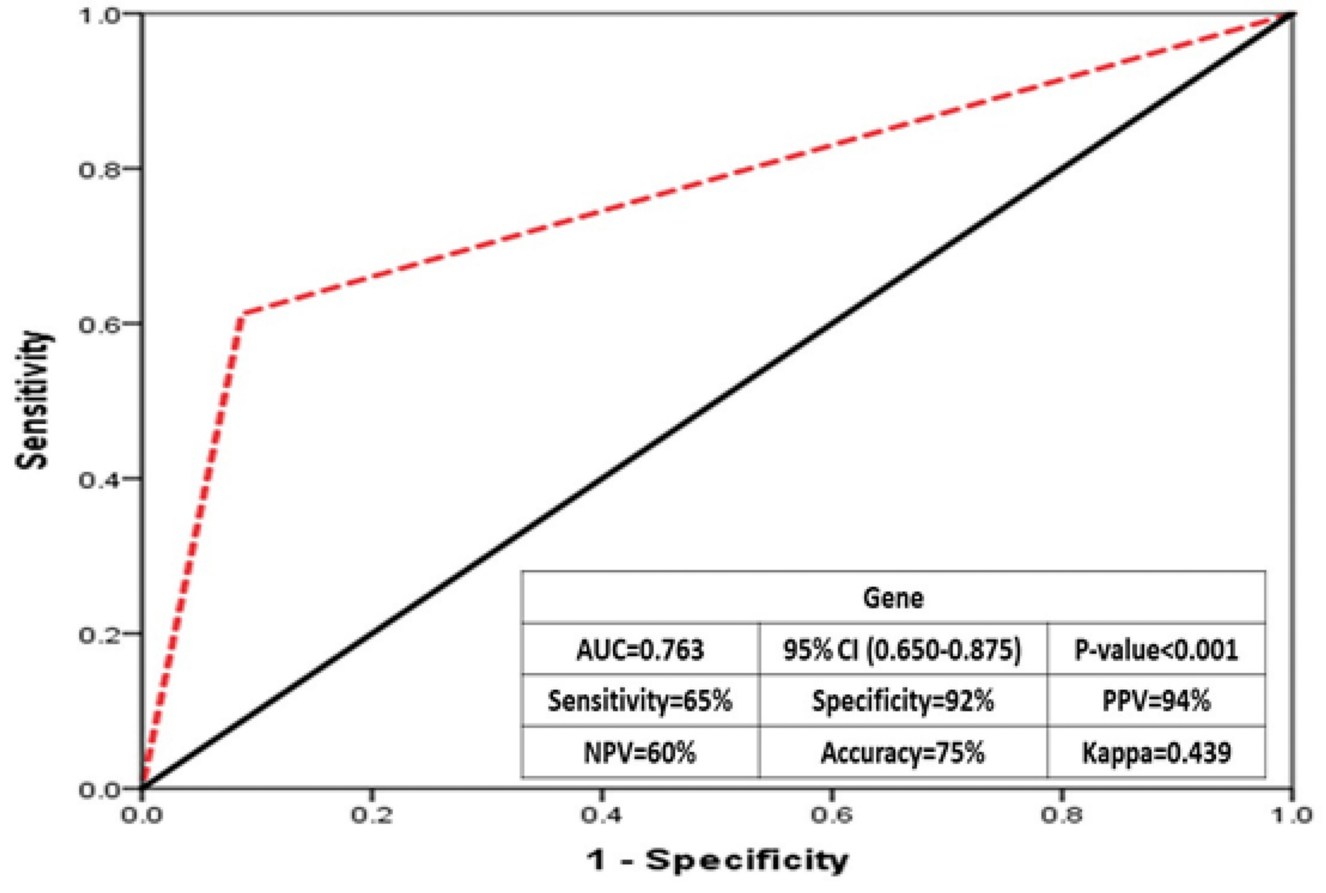
AUC for genes AUC: area under the curve; PPV: positive predictive value; NPV: negative predictive value AUC:=0.763; ^a ^standard error= 0.057; ^b^ 95% confidence interval= 0.650 to 0.875; Z statistic=4.682; Significance level P (Area=0.5)  < 0.001;  Youden’s index 0.57

Figure 3 shows the area under the receiver operating characteristic (ROC) curve (AUC) for gene mutations, the gene study's adjusted overall correlation (AUC) was 0.763, with 65% sensitivity, 92% specificity, 94% positive predictive value (PPV), 60% negative predictive value (NPV), and 75% overall accuracy, indicating that the gene study has a good confirmatory value for thalassemia screening.

## Discussion

The high frequency of beta thalassemia carriers and the rising number of newly diagnosed ones highlight the importance of implementing Egypt's program for preventing beta thalassemia. Symptomatic thalassemia syndromes are a substantial public health issue in Egypt [2].

Our estimated thalassemia trait prevalence in close relatives of patients is consistent with El-Beshlawy et al.'s study, in which a carrier rate of 9-10.2% was estimated among 1000 randomly selected normal people from various Egyptian geographic locations [6]. The prevalence in the current study was also very comparable to that reported by Ahmed et al. in Rawalpindi, Pakistan (31%) [14]. However, it was greater than the prevalence seen in extended family screening in India, as reported by Gorakshakar and Colah (21.9%) [15], and lower than the results of the studies conducted in Faisalabad (44.4%) [16], Kota, India (48.76%) [17], Bandung (59.6%) [18], Karachi (62.2%) [19], Bhopal (76%) [20], and North India (76.92%) [21]. The disparity between these studies, findings could be attributed to the genetic heterogeneity of the thalassemia gene, variations in the overall prevalence rates in the pertinent geographic areas, the selection criteria (extended family or siblings alone, the number of participants, consanguineous marriage, and rural residence), and the studies' selection criteria.

Our study results found a statistically significant difference between patients from rural and urban areas, with a significant increase in beta thalassemia carriers among those living in rural areas. Our results were also consistent with those of Maskoen et al., who conducted a study on 196 participants from close relatives of beta thalassemia major cases in Indonesia and reported that the presence of rural areas in governorates may be responsible for the notable differences between them [18].

Regarding the laboratory data from the current study, carriers (children with the beta-thalassemia trait) had an extremely higher mean RBC count and serum ferritin level than non-carriers, and their mean levels of Hb, MCV, and MCH were lower.

As regards gene screening, our study results found that in 61.5% of our participants, screening for beta thalassemia mutations using ARMS-PCR, which covered the most common 22 alleles in the Mediterranean region, was successful in detecting the beta-globin genotype, where we detected the presence of 14 b-globin mutations with a detection rate of 63.6% (as we covered 22 alleles only from > 200 alleles responsible for thalassemia mutation) where the most common mutation was IVS 1.6 (T>C) (31.3%), followed by IVS 1.110 (G>A) (21.9%), and then IVS 1.1 (G>A), and IVS 2.848 (C>A), both of them 9.4%, followed by the rarest gene mutations: IVS 2.745 (C>G), Codon 39, and Codon 8 (-AA) (6.3% each), and then all the remaining genes: -30 (T>A), -87 (C>G), Codon 5 (-CT), Codon 27 (G>t), IVS 2.1 (G>A), IVS 1.5 (T>C), and lastly codon 44 (-C) (3.1% each).

Similar to our results, El-Gawhary revealed beta thalassemia mutation in 80 of the 95 cases from Fayoum; IVS I.6 (T>>C) was the most prevalent allele in the sample (36.3%) and IVS I.110 (G>>A) was the second-most frequent mutation (25.8%) [22]. In a similar study, Hussein et al. used genomic PCR and a range of mutation-screening techniques to discover the spectrum of mutations that cause beta thalassemia in Egypt in which IVS 1.110 (41%), IVS 1.6, IVS 1.1, and IVS 2.848 (11%) were found as the four mutations that comprised 78% of the beta thalassemia genes [23]. In another study by Hussein et al., IVS 1.110 (G>A) (31.4%) was the most prevalent mutation among patients from the Suez Canal area [24].

The findings of Elmezayen et al., who examined 47 Egyptian patients in total, including 25 men and 22 women who had been diagnosed with thalassemia, to map the range of mutations that lead to beta thalassemia on the Egyptian north coast, were consistent with our findings in that they also identified 10 globin mutations; IVS 1.6 (T>C) (27.66%), and IVS 1.110 (G>A) (22.35%) were the most common. Twenty-four patients (51%) had homozygous mutations, while 13 patients (28%) had compound heterozygous mutations [25].

In order to determine the range of mutations that cause beta thalassemia, El-Shanshory et al. examined 200 children with the disease in Egypt. using genomic PCR and several mutation-screening tools [26]. They found that the mutations IVS 1.110 (G>A) (48%), IVS 1.6 (T>C) (40%), and IVS 1.1 (G>A) (24%, were the most common. According to Omar et al.'s study in Alexandria, which included 50 transfusion-dependent beta thalassemia cases, the most prevalent mutation was IVS 1.110, followed by IVS 1.6 and IVS 1.1. None of the patients in this study had the IVS 1.1 or Cd-39 mutations [20].

Despite the novelty of gene studies in the confirmation of the diagnosis of thalassemia carriers, HPLC is still the gold standard in the diagnosis of thalassemia in Egyptian children.

Limitations of the study

This study was done on only 22 alleles suspected to be prevalent in our society but we recommend other researchers to use extended gene study for all known genes related to the occurrence of this disease or rapid exome sequencing in a consanguineous population [27]. Also, this study was done on 109 children, and we recommend a larger sample size for a more accurate determination of the prevalence of carriers of this disease

## Conclusions

Beta thalassemia trait is significantly prevalent among relatives of thalassemia children, and the most prevalent mutation in these children was IVS 1.6 (T>C). In spite of the fact that HbA2 is still the gold standard and a more reliable test for the diagnosis of thalassemia, gene study plays a very important role in the confirmation of the diagnosis of equivocal cases and the detection of the genetic defect in such cases. An extended gene panel is needed for a proper diagnosis of cases and carriers of thalassemia and the identification of complete genetic defects in such cases.
